# Baseline Brain Gray Matter Volume as a Predictor of Acupuncture Outcome in Treating Migraine

**DOI:** 10.3389/fneur.2020.00111

**Published:** 2020-03-05

**Authors:** Xue-Juan Yang, Lu Liu, Zi-Liang Xu, Ya-Jie Zhang, Da-Peng Liu, Marc Fishers, Lan Zhang, Jin-Bo Sun, Peng Liu, Xiao Zeng, Lin-Peng Wang, Wei Qin

**Affiliations:** ^1^Engineering Research Center of Molecular and Neuro Imaging of Ministry of Education, School of Life Science and Technology, Xidian University, Xi'an, China; ^2^Beijing Key Laboratory of Acupuncture Neuromodulation, Acupuncture and Moxibustion Department, Beijing Hospital of Traditional Chinese Medicine, Capital Medical University, Beijing, China; ^3^Institute of Acupuncture and Moxibustion, China Academy of Chinese Medical Sciences, Beijing, China; ^4^Department of Neurology, Beth Israel Deaconess Medical Centre and Harvard Medical School, Boston, MA, United States

**Keywords:** migraine, acupuncture, prediction, gray matter, machine learning

## Abstract

**Background:** The present study aimed to investigate the use of imaging biomarkers to predict the outcome of acupuncture in patients with migraine without aura (MwoA).

**Methods:** Forty-one patients with MwoA received 4 weeks of acupuncture treatment and two brain imaging sessions at the Beijing Traditional Chinese Medicine Hospital affiliated with Capital Medical University. Patients kept a headache diary for 4 weeks before treatment and during acupuncture treatment. Responders were defined as those with at least a 50% reduction in the number of migraine days. The machine learning method was used to distinguish responders from non-responders based on pre-treatment brain gray matter (GM) volume. Longitudinal changes in GM predictive regions were also analyzed.

**Results:** After 4 weeks of acupuncture, 19 patients were classified as responders. Based on 10-fold cross-validation for the selection of GM features, the linear support vector machine produced a classification model with 73% sensitivity, 85% specificity, and 83% accuracy. The area under the receiver operating characteristic curve was 0.7871. This classification model included 10 GM areas that were mainly distributed in the frontal, temporal, parietal, precuneus, and cuneus gyri. The reduction in the number of migraine days was correlated with baseline GM volume in the cuneus, parietal, and frontal gyri in all patients. Moreover, the left cuneus showed a longitudinal increase in GM volume in responders.

**Conclusion:** The results suggest that pre-treatment brain structure could be a novel predictor of the outcome of acupuncture in the treatment of MwoA. Imaging features could be a useful tool for the prediction of acupuncture efficacy, which would enable the development of a personalized medicine strategy.

## Introduction

Migraine is characterized by recurrent episodes of severe headaches that are often unilateral and are accompanied by symptoms of autonomic nervous system dysfunction, such as nausea, vomiting, photophobia, and phonophobia (1). Migraine ranks second among the global level-4 causes of disability and is the most common cause of disability in those aged 15–49 years (2, 3). Migraine was reportedly experienced by as many as 1.04 billion people in 2016 (2). Thus, there is a pressing need to improve the clinical care of migraine.

Acupuncture is used for migraine treatment in many countries (4–11), although its superiority compared with sham acupuncture and medication remains controversial (7, 9, 10). However, about 50% of patients do not achieve substantial improvement after acupuncture (10, 11). The ability to predict the efficacy of acupuncture would prevent non-responders from enduring a long period of unsuccessful treatment. In one previous study, the outcome of acupuncture in patients with migraine was predicted by the presence of throbbing symptoms and expectations for a cure (12); however, an objective prognostic biomarker is still lacking.

Two recent studies that used baseline brain structure to predict the placebo response of sham acupuncture in patients with migraine found that the baseline prefrontal cortex volume and the fibers of the prefrontal-amygdala region predicts the placebo outcome after 8 weeks and discriminate responders from non-responders (13, 14). This suggests the potential of neuroimaging markers as predictors of migraine acupuncture treatment outcomes. In addition, previous studies have found that patients with migraine have brain gray matter (GM) abnormalities. Patients with migraine without aura (MwoA) have increased GM in the thalamus, parahippocampal gyrus, and frontal gyrus regions (15–17), and have decreased GM in the brainstem region (18). These findings indicate that brain GM might be useful in predicting the response of MwoA to acupuncture treatment.

The machine learning classification method has been increasingly used to classify subtypes of patients or to predict remission and non-remission with certain treatments (19–21), thus providing a new strategy for the development of personalized treatment. The present study aimed to use machine learning technology to predict the responders to acupuncture treatment for MwoA based on pre-treatment brain GM volume. The longitudinal changes in the GM regions were also examined.

## Methods

### Participants

Chinese patients with MwoA were recruited from the outpatient acupuncture departments of the Beijing Hospital of Traditional Chinese Medicine, Capital Medical University between 2017 and 2019. The trial protocol was registered (ISRCTN11800433) and ethics approval was obtained from the Research Ethical Committee of Beijing Hospital of Traditional Chinese Medicine (ref: 2016BL-081-02) prior to trial commencement. The study was conducted in accordance with the principles of the Declaration of Helsinki. All patients provided written informed consent for study inclusion. One experienced neurologist assessed the eligibility of all potential participants following pre-defined inclusion/exclusion criteria and provided a detailed explanation of the study design. The inclusion criteria were MwoA diagnosed in accordance with the International Classification of Headache Disorders (versions III beta) (1); age 18–65 years; history of migraine for at least 1 year; migraine frequency attacks of more than twice a month; no prophylactic treatments with acupuncture or pharmacological medicine administered in the past 3 months; right-handedness.

The exclusion criteria were as follows: (1) chronic migraine, tension-type headache, cluster headaches, or another primary headache; (2) secondary headache or other neurological diseases, such as headache caused by otorhinolaryngology diseases or intracranial pathological changes, and a history of depression, Parkinson's disease, or other extrapyramidal diseases; (3) relatively severe systemic diseases (cardiovascular disease, acute infectious disease, hematopathy, endocrinopathy, allergy or methysis); (4) pregnancy or lactation; (5) use of prophylactic migraine medication in the last 3 months; (6) magnetic resonance imaging (MRI) contraindications such as cardiac pacemakers or other metallic implants; or (7) alcohol or drug abuse.

**Figure 1:**
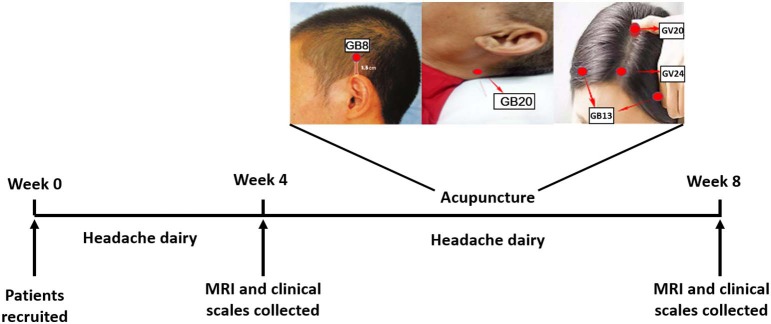
Experimental flow chart. The stimulation points were GV20 (Baihui), GV24 (Shenting), and bilateral GB13 (Benshen), GB8 (Shuaigu), and GB20 (Fengchi).

Participants were randomly divided into the acupuncture group and the sham acupuncture groups. This present study consisted of two phases: a baseline period after enrollment (week 1 to week 4) and a treatment period (week 5 to week 8). The experimental design is shown in Figure 1. Participants were required to keep a headache diary from the baseline period to the end of treatment. Imaging and clinical data were collected at the end of week 4 and week 8.

### Clinical Assessment

In the headache diaries, the participants recorded the details of their migraine attacks, including migraine days, intensity, locations, cause of the headache, concomitant symptoms (nausea, vomiting, photophobia, and phonophobia), and acute medications (if any) taken for each migraine attack. Headache intensity was assessed by the visual analog scale (VAS, 0 to 10). Participants who achieved at least a 50% reduction in the number of migraine days were defined as responders (10, 11, 14). Repeated-measures analysis of variance was used to analyze the changes in clinical data in responders vs. non-responders.

### Acupuncture Treatment

All participants received three acupuncture sessions each week for 4 weeks. Each session lasted for 30 min. Participants were allowed to take acute headache medication during this study and were required to record the details. The acupuncture points were selected based in accordance with information collected from a vast number of Chinese medicine reference books and the consensus of acupuncture experts based on their clinical experiences, and comprised GV20 (Baihui), GV24 (Shenting), bilateral GB13 (Benshen), GB8 (Shuaigu), and GB20 (Fengchi) (Figure 1). The sham acupuncture points are shown in the Methods in Supplementary Materials. Eight sterile disposable steel needles (gauge and size: 0.25 mm × 25 mm; Hwato Needles, Suzhou, China) were used in each acupuncture session. To ensure treatment consistency, all treatments were performed by one acupuncturist, who was registered with the Ministry of Health of the People's Republic of China and had more than 20 years of clinical experience.

### Imaging Data Acquisition

MRI was performed during the interictal headache phase, at least 3 days from last attack. Images were obtained using a 3-T Siemens MRI system (Skyra, Siemens Medical System, Erlangen, Germany) at the Beijing Hospital of Traditional Chinese Medicine, Capital Medical University. The parameters were as follows: voxel size 2.3 × 2.3 × 3.0 mm^3^, 40 continuous slices with a slice thickness of 3.0 mm, repetition time = 3,000 ms, echo time = 30 ms, flip angle = 90°, field of view = 220 × 220 mm, matrix = 94 × 94.

### Data Preprocessing

The structural image preprocessing and analysis were performed using Statistical Parametric Mapping12 (www.fil.ion.ucl.ac.uk), while the voxel-based morphometry analysis was performed using the Computational Anatomy Toolbox (CAT12) toolbox in the MatLab environment (www.mathworks.com). The CAT12 is an advanced and powerful qualitative MRI program that automatically evaluates the differences between regions with different GM volume without prior information to define the anatomical borders (22, 23). The Diffeomorphic Anatomic Registration Through Exponentiated Lie algebra algorithm normalization program included in the CAT12 toolbox was used to transform the structural magnetic resonance image of the native space into the 152 standard space template of the Montreal Neurological Institute. The images were segmented into white matter, GM, and cerebrospinal fluid to extract a GM region of 1.5 × 1.5 × 1.5 mm^3^ voxels. In the last step of the Diffeomorphic Anatomic Registration Through Exponentiated Lie algebra algorithm normalization program, the GM tissues were modulated by a non-linear deformation method to compare the relative GM volume after adjusting for individual brain size. After preprocessing, the CAT12 toolbox was used to perform a quality inspection to evaluate the homogeneity of the GM tissues. The normalized and modulated structure magnetic resonance images were then spatially smoothed with an 8-mm full-width at half-maximum Gaussian smoothing kernel.

### Feature Selection

Two-sample *t*-testing was first performed to identify brain voxels that had a significant difference in GM volume between responders and non-responders. Voxels with a *P*-value less than a specific number were selected. Considering the huge number of voxels in the GM template (1 × 1 × 1 mm), this number was set using the grid-search method from 0.0025 to 0.05 with a step of 0.0025. Clusters of at least 50 significant voxels were identified, and the average GM volume across the voxels in each cluster was extracted as the initial feature.

The 10-fold cross-validation-based least absolute shrinkage and selection operator (LASSO) method was then used to further shrink the initial features into fewer more important features. Briefly, datasets were randomly split into 10 groups. Each group was then excluded in turn, and the LASSO method with the mean squared error as the cost function was performed on the remaining nine groups (24). This step was repeated 10 times, resulting in 10 different sets of selected features. Finally, those features that occurred 10 times were selected as LASSO features for classification model construction.

### Model Construction

The linear support vector machine (SVM) method was used to construct the classification model based on the LASSO features. The accuracy, sensitivity, specificity, and dice similarity coefficient (DSC) were used as indices to assess the performance of the classification model. These four indices were defined as shown below:

(1) $$\begin{array}{r} \text{accuracy}=\;\frac{\text{TP}+\text{TN}}{\text{TP}+\text{TN}+\text{FP}+\text{FN}} \end{array}$$

(2) $$\begin{array}{r} \text{sensitivity}=\frac{\text{TP}}{\text{TP}+\text{FN}} \end{array}$$

(3) $$\begin{array}{r} \text{specificity}=\frac{\text{TN}}{\text{TN}+\text{FP}} \end{array}$$

(4) $$\begin{array}{r} \text{DSC}=\frac{2\text{TP}}{2\text{TP}+\text{FP}+\text{FN}} \end{array}$$

where TP represents true positive, TN represents true negative, FP represents false positive, and FN represents false negative. Ten-fold cross-validation was used to estimate the reliability of the model. Briefly, subjects were randomly divided into 10 groups. Each group was used in turn for testing, while the remaining nine groups were used for training. The feature selection and classification model construction steps were performed only for the training group, while the testing group was used to test the performance of the model. Finally, the mean standard difference of each index across 10 performances was calculated.

### Correlation Analysis and Longitudinal Changes

After performing the 10-fold cross-validation, all GM masks that contained the clusters corresponding to the selected LASSO features in each training group were added, the number of times that each voxel occurred in these masks was counted, and those voxels that occurred at least five times were reserved, as those voxels that occurred less than five times were considered to be reliant only on the specific splitting training group. Next, the average GM volume across the selected voxels in each cluster was extracted as the GM predictive regions. To further investigate the relationship of these predictive regions with acupuncture outcome, Pearson correlation testing was performed to assess the correlations between Δmigraine days (pre-treatment number of migraine days—post-treatment number of migraine days) and baseline GM volume of the predictive regions. The two-sample *t* test was used to compare the differences between the 10 predictive regions in responders vs. non-responders at baseline. Repeated-measures analysis of variance was performed to detect the longitudinal changes in GM volume in the predictive regions. For GM with an interaction effect of group × time, *post-hoc* analysis was used to detect the GM volume changes in different groups and at different time points. SPSS for Windows (version 18) was used to analyze the abovementioned comparisons with Bonferroni correction for multiple comparisons.

## Results

### Clinical and Demographic Information

Forty-one patients who underwent acupuncture treatment were included in the final clinical analyses. Details of the sham acupuncture group were provided in the Supplementary Materials (Results, Table S1). The responder rate in the acupuncture group was significantly higher than that in the sham acupuncture group (*P* = 0.007, Table S2).

In the acupuncture group, 19 responders (46%) achieved a 50% reduction in the number of migraine days, which was close to the incidences of responders reported in our previous study and the study by Diener et al. (10, 11). The baseline information did not significantly differ between responders and non-responders (Table 1). After acupuncture treatment, the responders had significantly fewer migraine days and a significantly lower VAS scale than non-responders (Table 2). The number of patients using acute headache medication, such as aminopyrine phenacetin or ibuprofen, did not significantly differ between responders and non-responders after acupuncture treatment (Table 2).

**Table 1:** Baseline demographics and clinical information of acupuncture responders and non-responders.

	**Responders (*n* = 19)**	**Non-responders (*n* = 22)**	***P***
Age, years (SD)	35.0 (10.4)	37.5 (11.87)	0.481^a^
Women, *n* (%)	13 (68.4)	20 (90.9)	0.157^b^
Duration of illness, year (SD)	15.3 (8.4)	14.7 (9.9)	0.854^a^
Days of migraine (SD)	6.9 (5.0)	9.0 (7.6)	0.310^a^
**LOCATION OF HEADACHE**, ***N*** **(%)**			
Unilateral	7 (36.8)	6 (27.3)	0.511^b^
Bilateral	12 (63.2)	16 (72.7)	
**CAUSE OF HEADACHE**, ***N*** **(%)**			
Tiredness	6 (31.6)	10 (45.5)	0.364^b^
Sleep problems	12 (63.2)	12 (54.5)	0.577^b^
Mental stress	13 (68.4)	13 (59.1)	0.536^b^
Other	15 (78.9)	13 (59.1)	0.173^b^
**ACCOMPANYING SYMPTOMS**, ***N*** **(%)**			
Nause or vomting	16 (84.2)	17 (77.3)	0.87^b^
Photophobia or audiaphobia	14 (73.7)	13 (59.1)	0.326^b^
Other	10 (52.6)	10 (45.5)	0.647^b^

*Data were presented as mean ± standard deviation (SD), number (percentage)*.

a *P-values based on the independent two sample t-test*.

b *P-values based on the chi-squared test*.

**Table 2:** Clinical outcome measures.

	**Time point**	**Responders (*n* = 19)**	**Non-responders (*n* = 22)**	***P***
Difference from baseline in days of migraine (SD)	Week 4	4.8 (3.7)	0.5 (2.6)	8.253 × 10^−5^^a^
Visual analog scale (SD)	Baseline	7.7 (1.6)	7.2 (1.5)	1.106 × 10^−4^^c^
	Week 4	4.0 (2.9)^***^	6.6 (1.6)^#^	
Number of people with acute medication, *n* (%)	Baseline	6 (31.6)	9 (40.9)	0.536^b^
	Week 4	6 (31.6)	9 (40.9)	0.536^b^

*Data were presented as mean ± standard deviation (SD), number (percentage)*.

a *P-values based on the independent two sample t-test*.

b *P-values based on the chi-squared test*.

c *P-values based on repeated measurement analysis of variance*.

*** *P < 0.001 for the post-hoc comparison of pre- vs. post-treatment values in responders*.

# *P < 0.05, for the post-hoc comparison of responders vs. non-responders in post-treatment values*.

### Classification Results

**Figure 2:**
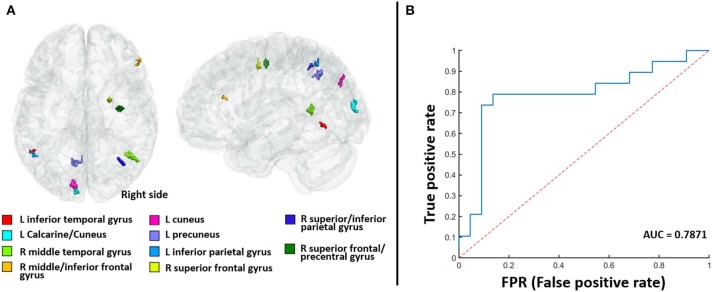
The 10 identified gray matter predictive regions **(A)** and the receiver operating characteristic curve of the classification model **(B)**. The area under the curve (AUC) is 0.7871.

The classification of responders and non-responders showed a high degree of precision (sensitivity 73%, specificity 85%, accuracy 83%, and DSC 75%). Fourteen participants were classified as responders (true value was 19) and 20 were classified as non-responders (true value was 22). Figure 2B displays the receiver operating characteristic curve of the classification model. The area under the curve was 0.7871. Together, these results demonstrate the stability of our classification model and the reliability of our selected features.

After counting the number of occurrences of each voxel corresponding to the selected LASSO features in 10-fold cross-validation, 10 GM predictive regions were finally defined. Figure 2A and Table S3 show the spatial distribution of these 10 GM predictive regions and the detailed regional information. Next, a Radiomics score (Rad-score) coefficient was constructed in accordance with the weight value of each GM predictive region in the linear SVM model (Supplementary Materials, Results) where a Rad-score of <0 represents a responder and a Rad-score of >0 represents a non-responder.

Baseline clinical features and imaging data were also combined to perform prediction analysis. Details were provided in the Supplementary Materials (Results).

### Correlation Analysis and Longitudinal Changes

**Figure 3:**
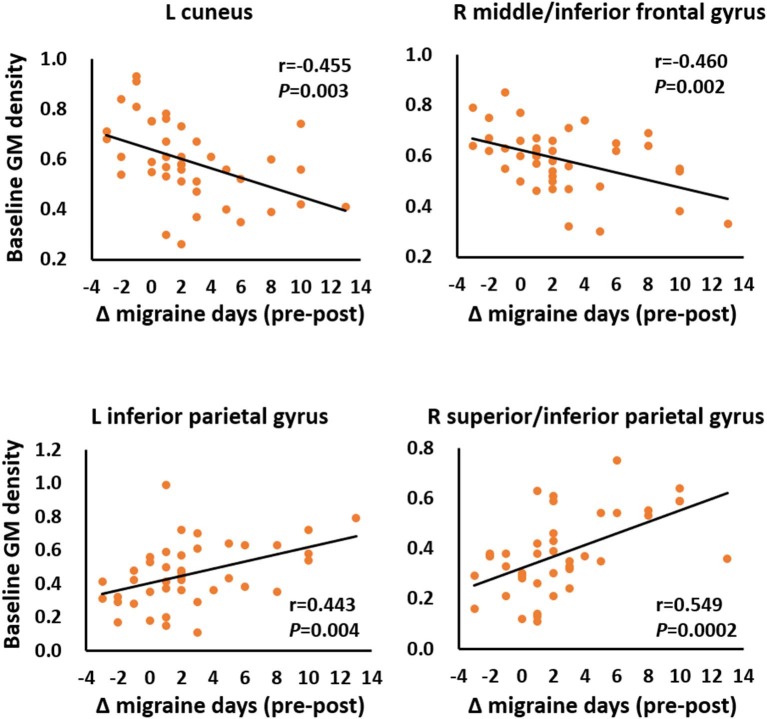
Correlations between the reduction in the number of migraine days (Δ migraine days) and the baseline gray matter (GM) volume of the predictive regions.

**Figure 4:**
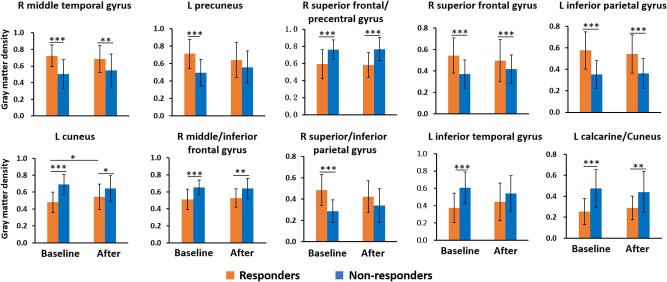
Longitudinal changes in gray matter volume among responders and non-responders. All predictive regions showed a significant difference in baseline gray matter volume between responders and non-responders. The left cuneus in responders showed a longitudinal increase in gray matter volume. L, left; R, right. *P < 0.05; **P < 0.01; and ***P < 0.001.

Among 10 predictive regions, the Δmigraine days in all patients was correlated with the baseline GM volume of four regions, including the left cuneus (*r* = −0.455, *P* = 0.003), right middle frontal/inferior frontal gyrus (*r* = −0.460, *P* = 0.002), left inferior parietal gyrus (*r* = 0.433, *P* = 0.004), and superior/inferior parietal gyrus (*r* = 0.549, *P* = 0.0002) (Figure 3). In addition, the baseline GM volume in all predictive regions significantly differed between responders and non-responders (Figure 4).

In the longitudinal analysis, the GM volume of the left cuneus showed a significant group × time interaction (*F* = 9.159, *P* = 0.004, Figure 4), in which the responders achieved an increase in GM volume after 4-weeks acupuncture treatment, while the non-responders did not. However, no correlation was found between the ΔGM volume of the left cuneus and the Δmigraine days in responders.

## Discussion

Treatment personalization is an important trend for the future in medicine. The use of medical imaging information to assist in disease diagnosis is being increasingly applied in the fields of cancer medicine (25) and psychology (19–21). However, few studies have used medical images to predict the efficacy of acupuncture. The present study used the machine learning classification method to establish a predictive model of acupuncture efficacy in patients with MwoA based on pre-treatment brain GM structure. The model had an 83% accuracy rate in distinguishing the acupuncture responders from the non-responders. These results provide an objective potential biomarker for the acupuncture treatment response of patients with migraine and also offer a new strategy for the development of personalized medicine for MwoA.

A common problem in traditional Chinese medicine is the individual differences in the efficacy of acupuncture. As shown in the present study, only about 50% of patients achieved substantial symptom improvement after 1 month of acupuncture treatment. Thus, the prediction of acupuncture response could reduce medical costs for patients identified as probable non-responders. Several previous studies have investigated the prediction of the outcome of migraine treatment. In patients with headache, genetic factors, migraine characteristics, and autonomic symptoms have been evaluated to predict treatment response to triptan and topiramate (26–28). In addition, white matter hyperintensity was found to predict migraine prognosis (29); however, the correlation and regression analysis between predictors and patient outcomes used in this previous study were insufficient. The present study used a linear SVM-based classification to distinguish between responders and non-responders. This method can be used to identify a hyperplane to separate the two groups by minimizing the empirical classification error in training data and achieving a higher degree of accuracy for unseen data, which enables the distinction of responders from non-responders at an individual level (30, 31). A previous study achieved an accuracy of 84% using a linear SVM to predict the outcome of acupuncture placebo treatment in patients with MwoA (13). In our model (83% accuracy), the baseline GM volume in 10 regions located in the temporal, frontal, parietal, cuneus, and precuneus gyri that differs between responders and non-responders at baseline was able to predict acupuncture efficacy in patients with MwoA. Previous neuroimaging studies have found that most of these regions were correlated with the pathophysiology of migraine. There is a marked decrease in the GM volume in the frontal, temporal, occipital, and precentral gyri in patients with migraine (32–34), and acupuncture might modulate the abnormal function of the frontal gyrus, temporal gyrus, precuneus, and cuneus in patients with migraine (35–37). Our study revealed the value of these regions in migraine acupuncture therapy from the perspective of individualized prognosis.

The present results also showed that the baseline GM volume in four regions was directly correlated with the acupuncture outcome. The efficacy of acupuncture was greatest in those patients with the lowest baseline GM volume in the middle frontal/inferior gyrus and cuneus and the greatest baseline GM volume in the parietal gyrus; this was consistent with the GM features in responders. At baseline, responders had less GM volume in the middle frontal/inferior gyrus and cuneus and greater GM volume in the parietal gyrus compared with non-responders. Combined with the contribution of these regions in the predictive model, the present results suggest that the baseline GM structure in these regions may play an important role in determining the clinical outcome of acupuncture treatment.

The GM volume of the left cuneus in responders had significant longitudinal change after 1 month of acupuncture treatment. A previous study revealed an increase in the regional function in the cuneus after acupuncture treatment in patients with MwoA (36). Therefore, the plasticity change of the cuneus may be involved in the mechanism in acupuncture efficacy in treating migraine disease. However, the present study and the study by Zhang et al. (36) did not identify a correlation between the structural or functional changes of the cuneus and the clinical efficacy of acupuncture for MwoA. In addition, the present study lacked healthy controls, and so it was not possible to assess whether the GM volume after acupuncture in responders was turned to the GM volume in healthy people. Therefore, it is unclear whether the post-treatment change in GM volume was beneficial. More studies are needed to determine whether acupuncture exerts its effects by regulating the GM structure or the function of the cuneus in responders.

The present study had several limitations. First, there were insufficient follow-up data collected because of a relatively high dropout rate, and so it was not possible to analyze the prediction of long-term outcome. The prediction of long-term acupuncture efficacy requires further study. Second, a large sample study of multimodal imaging information (cortical thickness, white matter structure, and brain function) should be considered as the next step in developing more precise and objective predictive models related to the outcome of acupuncture treatment. Third, the present study only included one dataset, and so the repeatability of the results cannot be verified. In the future, it is necessary to test the repeatability of the predictive model in more datasets related to acupuncture treatment of migraine, in order to establish a reliable predictive model that is helpful in clinical practice.

## Conclusion

With the increasing use of acupuncture therapy worldwide (38), the ability to predict the acupuncture outcome would contribute to the development of individualized treatment and promote its wider application. The current study used the machine learning classification method to establish a data-driven prediction model for acupuncture efficacy, which demonstrates that pre-treatment GM volume might be a novel biomarker for acupuncture outcomes in MwoA. In the future, MRI structure could be explored in more diseases to identify neuroimaging markers that predict the treatment response to acupuncture.

## Data Availability Statement

The datasets generated for this study are available on request to the corresponding author.

## Ethics Statement

The studies involving human participants were reviewed and approved by Research Ethical Committee of Beijing Hospital of Traditional Chinese Medicine (ref: 2016BL-081-02). The patients/participants provided their written informed consent to participate in this study. Written informed consent was obtained from the individual(s) for the publication of any potentially identifiable images or data included in this article.

## Author Contributions

X-JY contributed to the study design, data analysis, interpretation of the data, and drafting of the manuscript. LL contributed to the data acquisition and drafting of the manuscript. Z-LX contributed to the data analysis and drafting of the manuscript. Y-JZ contributed to the acquisition. LZ contributed to the data analysis. D-PL contributed to the data acquisition. MF critically revised the manuscript for intellectual content. J-BS, PL, and XZ contributed to the interpretation of the data. L-PW contributed to the study design and conceptualization and data interpretation. WQ contributed to the study design and conceptualization, data analysis, and interpretation.

### Conflict of Interest

The authors declare that the research was conducted in the absence of any commercial or financial relationships that could be construed as a potential conflict of interest.
