# Supplementary material for: Baseline Brain Gray Matter Volume as a Predictor of Acupuncture Outcome in Treating Migraine
Source: Front Neurol. 2020 Mar 5;11:111. doi: 10.3389/fneur.2020.00111 (PMC7066302; doi:10.3389/fneur.2020.00111)
Supplement: Supplementary file 1 [file Data_Sheet_1.pdf]

Supplemental Materials

Methods

The following stimulation points were used for sham acupuncture. Sham-point 1: in the medial aspect of the arm on the anterior border of the insertion of the deltoid muscle at the junction of the deltoid and biceps muscles; Sham-point 2: at the edge of the tibia, 1 - 2 cm lateral and horizontal to Zusanli (ST36); Sham-point 3: on the ulnar side of the arm, halfway between the epicondylus medialis of the humerus and the ulnar side of the wrist; Sham-point 4: halfway between the tip of the elbow and the axilla. These four points arelocated distant to traditionally recognized acupoints or meridians lines. The stimulation method was the same as that used in the acupuncture group.

Results

Clinical and demographic information

Fifty-five patients were orginally recruited in the acupuncture group, and Fifty-five were recruited in the sham acupuncture group. The number of recruited participants was slightly more than what was recorded in the ISRCTN registry system. The initial number of participants was determined based on our unpublished previous pilot study, in which the sample size was 45 in the acupuncture group and 45 in the sham acupuncture group. Considering an estimated 10% dropout, the original study protocol stated that a total of 100 participants would be enrolled, with 50 participants in each group. However, based on further advice from experts, the estimated dropout rate was changed from 10% to 20% due to the potential for poor image quality; therefore, our enrolment was expanded to a total of 110 participants (assuming a 20 % dropout rate). These changes were made prior to the trial commencement.

The final clinical analyses included 41 participants in the acupuncture group (seven were

excluded because of a lack of post-treatment images, four dropped out and three had poor quality images) and 39 in the sham acupuncture group (6 exited the study and 10 dropped out). There were no significant differences between the acupuncture and sham acupuncture groups in demographic and baseline clinical parameters (Table S1). After 4 weeks acupuncture treatment, both groups showed significant decreases in the VAS, with no significant differences between groups (Table S2). The acupuncture group had a significantly higher responder rate (*P* =0.007) and change in the number of migraine days (*P* = 0.022) than the sham acupuncture group (Table S2).

Table S1. Clinical and demographic information of all participants

	Acupuncture (n=41)	Sham (n=39)	*P*
Age, years (SD)	36.3 (11.1)	34.9 (9.0)	0.534 [a]
Women, n (%)	33 (80.5)	35 (89.7)	0.247 [b]
Duration of illness, year (SD)	15.0 (9.1)	13.4 (6.9)	0.376 [a]
Days of migraine (SD)	8.0 (6.5)	7.5 (4.3)	0.698 [a]
Location of headache, n (%)			
Unilateral	13 (31.7)	12(30.8)	0.928 [b]
Bilateral	28 (68.3)	27 (69.2)	
Cause of headache, n (%)			
Tiredness	16 (39.0)	9 (23.1)	0.124 [b]
Sleep problems	24 (58.5)	27 (69.2)	0.320 [b]
Mental stress	26 (63.4)	18 (46.2)	0.121 [b]
Other	28 (68.3)	29 (74.4)	0.549 [b]
Accompanying symptoms, n (%)			
Nause or vomting	33 (80.5)	28 (71.8)	0.361 [b]

Photophobia or audiaphobia	27 (65.9)	27 (69.2)	0.747 [b]
Other	20 (48.8)	24 (61.5)	0.252 [b]

Data were presented as mean ± standard deviation (SD), number (percentage).

[a] *P* values based on an independent two-sample t-test.

[b] *P* values based on the chi-squared test.

Table S2. Clinical outcome measures

	Time point	Acupuncture (n=41)	Sham (n=39)	*P*
Responder rate, n (%)		19 (46.3)	7 (17.9)	0.007 [b]
Difference from baseline in days of migraine (SD)	Week 4	2.5 (3.8)	0.7 (2.9)	0.022 [a]
Visual Analogue Scale (SD)	Baseline	7.4 (1.5)	7.6 (2.0)	0.483 [c]
	Week 4	5.4 (2.6) ***	5.9 (1.8)***	
Number of people with acute medication, n (%)	Baseline	15 (36.6)	17(43.6)	0.523 [b]
	Week 4	15 (36.6)	16(41.0)	0.684 [b]

Data were presented as mean ± standard deviation (SD), number (percentage).

[a] *P* values based on the independent two-sample t-test.

[b] *P* values based on the chi-squared test.

[c] *P* values based on repeated measurement analysis of variance.

***$P<0.001$ for the comparison within each group.

Classification results

Table S3. Characteristics of the 10 predictive regions

Predictors	Weight	Voxel
L inferior temporal gyrus	0.0804	21
L calcarine/cuneus	0.1242	56
R middle temporal gyrus	0.0999	120
R middle/inferior frontal gyrus	0.1136	20
L cuneus	0.1077	43
L precuneus	0.0953	68
L inferior parietal gyrus	0.1008	25
R superior frontal gyrus	0.0809	19
R superior/inferior parietal gyrus	0.0795	26
R superior frontal/precentral/gyrus	0.1177	33

Radiomics score (Rad-score) calculation formula：

$$\begin{aligned}\text{Rad-score} = {} & 0.8966 \times \text{L inferior temporal gyrus} + 1.3851 \times \text{L calcarine/cuneus} \\ & - 1.1143 \times \text{R middle temporal gyrus} \\ & + 1.2676 \times \text{R middle/inferior frontal gyrus} + 1.2014 \times \text{L cuneus} \\ & - 1.0628 \times \text{L precuneus} - 1.1241 \times \text{L inferior parietal gyrus} \\ & - 0.9029 \times \text{R superior frontal gyrus} \\ & - 0.8862 \times \text{R superior/inferior parietal gyrus} \\ & + 1.3133 \times \text{R superior frontal/precentral/gyrus}\end{aligned}$$

Where, a Radscore of < 0 represents a responder and a Radscore of > 0 represents a non-responder.

In addition to using baseline imaging data for outcome prediction analysis, the baseline clinical features and imaging data were also combined to perform prediction analysis. The baseline clinical features included age, sex, duration of illness, number of migraine days, headache intensity, location of headache, and number of participants taking acute medication. The results showed a high degree of precision (sensitivity 90%, specificity 75%, accuracy 88%, and DSC 83%). The area under the curve was 0.8182. Therefore, combining multimodal data to establish predictive models may contribute to improving predictive performance.

Supplementary Figures

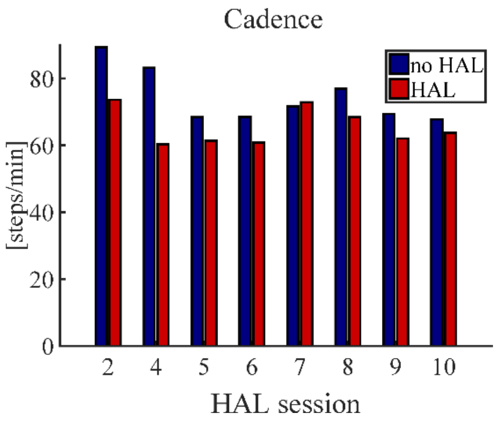

Supplementary Figure 1. Cadence during walking with HAL (HAL) and without HAL (NoHAL), in each of the HAL sessions.
